# The association of a single nucleotide polymorphism in the promoter region of the *LAMA1* gene with susceptibility to Chinese high myopia

**Published:** 2011-04-22

**Authors:** Yan Yan Zhao, Feng Ju Zhang, Si Quan Zhu, Hui Duan, Yang Li, Zhong Jun Zhou, Wen Xian Ma, Ning Li Wang

**Affiliations:** 1 Eye Center of Beijing Tongren Hospital of Capital Medical University, Beijing Ophthalmology & Visual Sciences Key Lab, Beijing, China; 2 Department of Ophthalmology, First affiliated Hospital of Dalian Medical University, Dalian, China; 3 Department of Biochemistry, Hongkong University, Hongkong, China

## Abstract

**Purpose:**

High myopia is a severe hereditary ocular disease leading to blindness. *LAMA1* (alpha subunit of laminin) is a promising candidate gene for high myopia present in the MYP2 (myopia 2) region. The purpose of this study was to determine if high myopia is associated with single nucleotide polymorphism (SNP) variants in *LAMA1* in Chinese subjects.

**Methods:**

Ninety-seven Chinese subjects with high myopia and ethnically and sexually matched 103 normal controls were enrolled. Genomic DNA was prepared from peripheral blood. The 5 SNPs of *LAMA1* were analyzed using PCR and SNaPshot. Allele frequencies were tested for Hardy–Weinberg disequilibrium. The genotype and allele frequencies were evaluated using the χ^2^ tests or the Fisher exact tests.

**Results:**

One of the 5 SNPs showed a significant difference between patients and control subjects (rs2089760: p_genotype_=0.005, p_allel_=0.003). There were no statistically significant differences between patients and control subjects for the other four SNPs: rs566655, rs11664063, rs607230, and rs3810046.

**Conclusions:**

Our results indicate that the polymorphism of rs2089760, located in the promoter region of *LAMA1*, may be associated with high myopia in the Chinese population and should be investigated further.

## Introduction

Myopia is a significant public health problem worldwide, with the highest prevalence in East Asians. The Handan eye study [1] showed the prevalence rate of myopia and high myopia (myopia in excess of 6 diopters [D])in a rural Chinese adult population was 26.7% and 1.8% separately, and a study of the Singapore adult Chinese population [2] showed the prevalence rate of myopia and high myopia was 38.7% and 9.1% separately. High myopia can cause blindness or a severe loss of visual acuity due to retinal detachment, submacular hemorrhage, glaucoma or macular degeneration [3], and 30% to 70% of high myopia display at least some lesions of the retina and choroids [4]. However, effective treatment methodology and preventive strategies for high myopia have not yet been fully established. Therefore, it is important to identify the etiology of high myopia.

Myopia is a complex disease involving multiple interacting genetic and environmental factors. Studies of twins provide the most compelling evidence that myopia is inherited. Multiple studies note an increased concordance of refractive error and refractive components (corneal curvature, lens power, anterior chamber depth) in monozygotic twins compared with dizygotic twins [5,6]. Twin studies estimate a notable heritability value (the proportion of the total phenotypic variance that is attributed to the genome) of between 0.5 and 0.96 [7].

In 1998, Young [8] performed a linkage analysis of eight families with high myopia in two or three successive generations, containing a total of 82 individuals to obtain the maximum lod score of 9.59 for the microsatellite marker D18S481. This region, which mapped at 7.6 cM on the short arm of chromosome 18 (18p11.31, MYP2 [myopia 2]), was indicated to be a susceptibility genetic locus for high myopia. Additionally, the MYP2 locus has been confirmed by two outside laboratories: an Italian patient population with autosomal dominant high myopia by Heath and colleagues [9] and six families of Hong Kong Chinese descent by Lam and colleagues [10]. All genes that map within the MYP2 critical region are candidate disease genes based on position. Coding regions, intron-exon boundaries and untranslated exons of Clusterin-like 1 (*CLUL1*), elastin microfibril interfacer 2 (*EMILIN2*), lipin 2 (*LPIN2*), myomesin 1 (*MYOM1*), myosin regulatory light chain 3 (*MRCL3*), myosin regulatory light chain 2 (*MRLC2*), transforming growth β-induced factor (*TGIFβ*), large Drosophila homolog associated protein 1 (*DLGAP1*), and zinc finger protein 161 homolog (*ZFP161*) were sequenced, but mutation analysis did not identify sequence alterations associated with high myopia [11]. The direct analysis of sequence within a critical region can be the most accurate, precise and efficient approach to disease gene identification. But susceptibility loci contributing to high myopia may be difficult to map by classic linkage analysis because of the limited power to detect genes of intermediate or small effect using independent pedigrees. Currently, genetic association studies are regarded as the most powerful approach to mapping of the genes underlying such complex traits [12]. And TGIF has been implicated as the MYP2-causative gene by single nucleotide polymorphism (SNP) association studies [13], but has not been replicated in a second Chinese case– control study [14] and a Japanese case– control study [15].

The identification of the MYP2 gene will not only provide insight into the molecular basis of high myopia, but will also identify pathways that are involved in eye growth and development. In addition, this information may implicate other genes as possible myopia disease gene candidates. Among all the MYP2 genes, laminin α chain (*LAMA1*) is a biologically relevant candidate gene, since laminin is a component of a structural glycoprotein found in the ocular scleral wall. Laminin is present in the eye as a constituent of the elastic system in the trabecular meshwork [16] and zonular (oxytalan) fibers of the lens [17]. It has also been identified in the astrocytic and vascular endothelial-cell basement membranes of the laminar-beam margins of the rodent lamina cribosa [18]. Marshall [19] has localized laminin to the oxytalan and elaunin microfibrils of human sclera by immunoelectron microscopy. These microfibrils comprise two of the three components of the elastic-fiber system that make elastic tissue more stretchable than collagen [20,21]. Marshall suggests that laminin may bind these microfibrils to collagen fibrils, since laminin has been shown to have binding sites for several extracellular matrix components, including collagen [22]. *LAMA1* gene was reported to be located at the short arm of chromosome 18 [23], approximately 1,648 kb centromeric of the *ZFP161* gene in the MYP2 region, *LAMA1* attracts our attention as a promising candidate gene for high myopia. Recently, we found the mRNA level of *LAMA1* was lower in high myopic scleral tissue than in non-myopic scleral tissue through testing the transcriptional level (mRNA level) of *LAMA1* in scleral tissue [24].

Here, to further investigate the correlation between *LAMA1* and high myopia, we conducted a case-control study to analyze the SNPs of *LAMA1* for association with high myopia.

## Methods

### Subjects

A total of 97 patients were enrolled: 39 males, 58 females; mean age of 40.4±12.3 years; refractive error: −6.00 D or more negative and ocular axial lengths: more than 26 mm for both eyes. One hundred-three unrelated control subjects were enrolled: 43males, 60 females; mean age of 45.8±13.5 years; refractive errors: −1.00 D to 1.00D and ocular axial lengths: 22 mm to 24 mm for both eyes. Auto refraction (auto keratometer, ARK 700A; Topcon, Tokyo, Japan) was performed on both eyes of each patient by experienced optometrists who were trained and certified in the study protocols. Corneal curvature (average of K1 and K2), anterior chamber depth (ACD)and axial length measurements were presented in Table 1. Comprehensive ophthalmic examinations were performed, and blood samples were collected from all patients. None of the participants had a history of ocular disease or ocular insult that may affect an individual’s refraction, such as retinopathy of prematurity or neonatal ocular problems or a genetic disease or connective tissue disorder associated with myopia, such as Stickler or Marfan syndrome. Clinical examination included visual acuity, refractive error, slit lamp examination, ocular movements, intraocular pressure, and fundus examination. Patients with organic eye disease; a history or evidence of intraocular surgery; and/or a history of cataract, glaucoma, retinal disorders, or laser treatment were excluded.

**Table 1:** Refraction status and ocular biometric measures of participants.

**Characteristics**	**Patients**		**Control**	
** **	**Right eye**	**Left eye**	**Right eye**	**Left eye**
K1 (mm) (Mean)	7.83	7.82	7.75	7.74
Std.	0.36	0.33	0.24	0.23
K2 (mm) (Mean)	7.59	7.60	7.57	7.60
Std.	0.35	0.32	0.24	0.24
ACD (mm) (Mean)	3.51	3.52	3.11	3.12
Std.	0.44	0.45	0.45	0.46
Axial length (mm) (Mean)	29.79	29.69	23.19	23.24
Std.	2.97	2.91	0.59	0.62
spherical equivalent (D) (Mean)	−13.81	−13.07	−0.47	−0.13
Std	6.10	5.63	0.54	−0.69

ACD: anterior chamber depth ; K: keratometry value (K1 the power in the meridian with greatest curvature and K2 the power in the meridian perpendicular to it).

This study was approved by the ethics committee of Dalian Medical University, Dalian, China, and informed consent was obtained from all patients. The study was performed according to the tenets of the Declaration of Helsinki for research involving human subjects.

### DNA extraction

Total genomic DNA was extracted from 10 to 15 ml of venous blood from all participants, after informed consent was obtained. DNA was purified from lymphocyte pellets according to standard procedures using a kit (Puregene kit; Gentra Systems, Minneapolis, MN).

### SNP selection

We used the NCBI dbSNP database to extract the available information of the SNPs in *LAMA1*. SNPs were selected using criteria such as population-frequency validation, multiple submitters and high-profile submitters. The most important criteria was selecting those likely to alter *LAMA1* transcription or translation. So we focused on the SNPs located in exons or 5′-flanking or UTR regions. A total of 5 SNPs used in this study were: rs2089760 in the 5′-flanking region, rs566655 in exon 14, rs11664063 in exon 39, rs607230 in exon 42, and rs3810046 in the 3′-flanking region.

### Analysis of *LAMA1* polymorphisms

Single nucleotide polymorphisms (SNPs) were determined by multiplex SNaPshot technology (according to previously described methods [25-27], using an ABI fluorescence-based assay allelic discrimination method (Applied Biosystems, Bedford, MA).The primers for polymerase chain reaction (PCR) amplification (shown in Table 2) and SNaPshot extension reactions (shown in Table 2) were both designed to be aligned with the NCBI sequence databases using Primer3 software. The extension primer was designed to anneal immediately adjacent to the nucleotide at the mutation site, either on the sense or antisense DNA strand. PCR was performed in a total volume of 10 μl containing 1× HotStarTaq buffer 1 μl, 3.0 mM Mg^2+^, 0.3 mM each of dATP, dCTP, dTTP, and dGTP, 1 U HotStarTaq polymerase (Qiagen, Chatsworth, CA), 1 μl genomic DNA, and 1 μl of each primer. The samples were put through 30 to 40 cycles of denaturation at 95 °C, annealing at specific primer temperatures, elongation at 72 °C, and a final extension at 72 °C. The PCR product was purified by 1 U SAP and 1 U Exonuclease I. The product was then processed according to the ABI SNaPshot protocol. Extension was performed in a total volume of 10 μl containing 5 μl SNaPshot Multiplex Kit (ABI), 2 μl PCR product,1 μl mixed extension primer and 2 μl H_2_O. The samples were put through 28 cycles of denaturation at 96 °C, annealing at 50 °C, elongation at 60 °C, and a final extension at 72 °C. The extension product was purified by 1 U SAP (shrimp alkaline phosphatase). SNP analysis was performed using an ABI3130 genetic analyzer. Genotypes were determined automatically using Genemapper4.0 software (Applied Biosystems).

**Table 2:** *LAMA1* sequence variants and PCR and extension primers.

**RefSNP** **ID**	**SNP Property**	**Base Pair** **Change**	**Amino acid Change**	**PCR primer**
rs2089760	5′-flanking	G>A	/	F: TGCATCCTTTTAAAACGGCCAAA
				R: TTTCCTCTCACTTGTGTGAATCTATTTGA
rs566655	non-synon (exon 14)	A>C	p.Asn674Thr	F:CGTGACCAGCTGATGACTGTCC
				R:CAATACATACCTGTAAAGAGCCATTTTTGC
rs11664063	non-synon exon 39)	G>A	p.Ala1876Thr	F:GTCTGCCAAAATCAGGCACCAC
				R:TTCCCACAAAGGCGTGTTCCTA
rs607230	non-synon exon 42)	A>G	p.Lys2002Glu	F:CCAGGCAAACCAATGAATCACTC
				R:CCTTTGCAAGTAAAAATTTTGCCAATC
rs3810046	3′-flanking	A>C	/	F:TCCAATTTCTACAACAGACAAGCAATG
				R:TGCAAAATGCGCTGTTAGGTGA
**Extension primer**				
rs2089760 SR	TTTTGTGTGAATCTATTTGACAACTCTATCAATT			
rs566655 SF	TTTTTTTTTTTTTTTTTTTTTTGACACATCTTTTGATCAGAGCCA			
rs11664063 SF	CAGAGCTGAGGACCATGCC			
rs607230 SR	AGAAGAAAGTCCTTTCCACTTACCTT			
rs3810046 SF	TTTTTTTTTTTTTTTTTTTTTTCAGACAAGCAATGTTCATTGATTAATT			

Rs:public reference SNP number from the dbSNP database; p: protein; non-synon: Non-Synonymous.

### Statistical analysis

We evaluated the allele frequencies of sequence alterations in patients and controls using the χ^2^ tests or the Fisher exact tests. All detected SNPs were assessed for Hardy–Weinberg disequilibrium using the χ2 test. Statistical analyses were performed on computer using the SPSS software (version 13.0; SPSS Science, Chicago, IL).The statistical between-group differences were examined using the respective allele models of dominant and recessive. A p<0.05 was considered statistically significant. The more powerful false discovery rate (FDR) [28], instead of the conventional Bonferroni procedure, was used to control for multiple hypothesis testing. Odds ratios (ORs) were calculated from genotype and allelic frequencies with a 95% confidence interval (CI).

## Results

**Figure 1:**
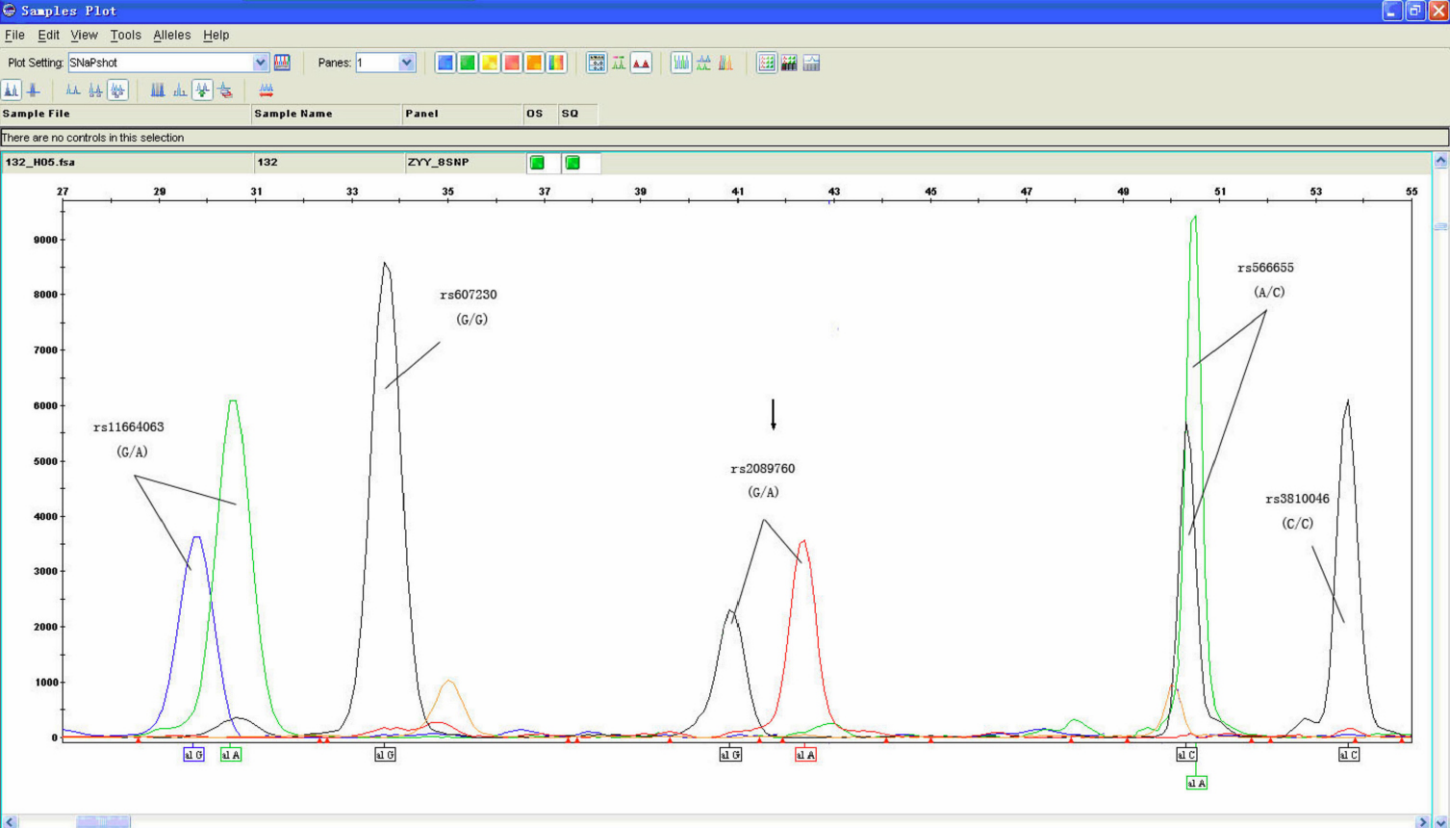
Multiplex SNaPshot analysis of 5 SNPs of *LAMA1*. Arrow: a heterozygote for the rs2089760 polymorphism.

We screened 5 SNPs within *LAMA1* for all the cases and controls (Figure 1). No deviations from Hardy–Weinberg equilibrium were observed (Table 3).The genotype distributions and allele frequencies of the five polymorphisms were shown in Table 3. Comparison of the genotypes between individuals with high myopia and the control group revealed no significant difference for four of five polymorphisms, including rs566655, rs11664063, rs607230, and rs3810046. However, one polymorphism in the 5′-flanking region (rs2089760) showed significant difference between the patients and the controls (Genotype: p=0.005; Allele: p=0.003 and OR: 1.378). After FDR correction, they were still significant (Genotype: p=0.033; Allele: p=0.03).

**Table 3:** Genotype and Allele frequencies for the 5 SNPs in *LAMA1*.

RefSNP ID	**Genotype frequencies**									**Allele frequencies**							
** **	**Patients**				**Control**				**p**	**Patients**		**Control**		p*	**OR**	**95% CI**	
** **	**1/1**	**1/2**	**2/2**	**HWD χ2**	**1/1**	**1/2**	**2/2**	**HWD χ2**		**Allel1**	**Allele2**	**Allel1**	**Allele2**			**low**	**high**
rs2089760	17 (17.5)	51 (52.5)	29 (30.0)	0.264	39 (37.9)	44 (42.7)	20 (19.4)	0.746	0.005	85 (43.8)	109 (56.2)	122 (59.2)	84 (40.8)	0.003	1.378	1.121	1.693
rs11664063	1 (1.0)	24 (24.7)	72 (74.3)	0.356	3 (2.9)	33 (32)	67 (65.1)	0.161	0.298	26 (13.4)	168 (86.6)	39 (18.9)	167 (81.1)	0.139	** **	** **	** **
rs607230	7 (7.2)	22 (22.7)	68 (70.1)	2.677	2 (1.9)	26 (25.3)	75 (72.8)	0	0.194	36 (18.6)	158 (81.4)	30 (14.6)	176 (85.4)	0.346	** **	** **	** **
rs3810046	3 (3.1)	36 (37.1)	58 (59.8)	0.659	3 (2.9)	31 (30.1)	69 (67)	0.012	0.564	42 (21.6)	152 (78.4)	37 (18)	169 (82)	0.381	** **	** **	** **
rs566655	70 (72.2)	26 (26.8)	1 (1)	0.420	65 (63.1)	32 (31.1)	6 (5.8)	0.228	0.122	166 (85.6)	28 (14.4)	162 (78.6)	44 (21.4)	0.09	** **	** **	** **

1/1: genotype with homozygous allele 1; 1/2: genotype with heterozygous alleles; 2/2b: genotype with homozygous allele 2; HWD indicates a χ2 statistic for Hardy–Weinberg disequilibrium. The genotype p value is based on a χ^2^ test. OR: odds ratio; 95%CI: 95% confidence interval. *Fisher exact tests.

In relation to the two types of alleles that are present at each SNP (allele 1 and allele 2), statistical between-group differences were examined using their respective allele models of dominant and recessive (shown in Table 4). For Allele1 recessive model (that allele 1 is present on both of the two homologous chromosomes, ie, in this model the disease does not occur unless homozygous for allele 1, this is implied to be also a dominant model for allele 2) only rs2089760 showed significant difference between the patients and the controls (p=0.002, after FDR correction p=0.04). For Allele1 dominant model (that allele 1 is present on either of the two homologous chromosomes, in this model the onset of disease occurs even when heterozygous for allele 1, this is implied to be also a recessive model for allele 2) no significant difference for the five SNPs.

**Table 4:** Genotype frequencies (Allele1 dominant and recessive model) for the 5 SNPs in *LAMA1*.

**RefSNP ID **	**Allele1 dominant model**					**Allele1 recessive model**				
** **	**Patients**		**Control**		p*	**Patients**		**Control**		p*
** **	**1/1+1/2**	**2/2**	**1/1+1/2**	**2/2**		**1/1+1/2**	**2/2**	**1/1**	**1/2+2/2**	
rs2089760	68 (70.1)	29 (29.9)	83 (80.6)	20 (19.4)	0.101	17 (17.5)	80 (82.5)	39 (37.9)	64 (62.1)	0.002
rs11664063	25 (25.8)	72 (74.2)	36 (35)	67 (65)	0.170	1 (1.0)	96 (99)	3 (2.9)	100 (97.1)	0.622
rs607230	29 (29.9)	68 (70.1)	28 (27.2)	75 (72.8)	0.754	7 (7.2)	90 (92.8)	2 (1.9)	101 (98.1)	0.093
rs3810046	39 (40.2)	58 (59.8)	34 (33)	69 (67)	0.307	3 (3.1)	94 (96.9)	3 (2.9)	100 (97.1)	1.0
rs566655	96 (99)	1 (1)	97 (94.2)	6 (5.8)	0.120	70 (72.2)	27 (27.8)	65 (63.1)	38 (36.9)	0.178

*Fisher exact tests.

## Discussion

The sclera, the tough outer coat of the eye, is a typical connective tissue that provides the structural framework for the eye. The sclera comprises extracellular matrix (ECM) and matrix secreting fibroblasts. It is arranged in layers (lamellae) that may play an important role in controlling the size of the eye. The extracellular matrix of the sclera has been shown to contain collagen fibrils in close association with proteoglycans and glycoproteins [29]. Alterations in any of these extracellular matrix components are likely to lead to changes in eye shape. Studies have shown that the scleral extracellular matrix undergoes significant changes during growth and aging [30] and is dramatically altered during the development of myopia [31,32]. Many of the pathological changes seen in highly myopic human eyes are a consequence of gross scleral thinning, particularly at the posterior pole of the eye [33]. It is presumed that a blurred image or light projected onto the retina induces secretion of some substance from cells, which evokes transfer of the signal to the sclera, thus leading to sclera remodeling. Laminin is a glycoprotein of 900 kDa with multiple domain structures. Its three component chains, α, β, and γ are bonded to each other with the α chain being in the central region and they form a cruciform structure [34]. Laminin is a component of an extracellular matrix protein that binds microfibrils to collagen fibrils, so *LAMA1* is a biologically relevant MYP2 candidate gene. And *LAMA1* maps to the18p11.31 region, its genome size is 175,928 bp composed of 62 exons, and the mRNA size of *LAMA1* is 9,530 bp encoding 3,075 amino acids [35,36].

While Sayaka Sasaki’s research in 2007 [37] found no statistically appreciable differences through analysising 13 SNPs of *LAMA1* through association study. But, we found their controls including cases with moderate myopia (refractive error weaker than −4.0 D), we thought it may influence the consequence. While our entry criteria was strict: patients with <−6.00 diopters for both eyes and ocular axial lengths of >26 mm for both eyes, controls with refractive errors of >-1.00 and <1.00 diopters for both eyes and ocular axial lengths of >22 mm and <24 mm for both eyes. Furthermore, we selected the cases with the normal corneal curvature [38,39] and ACD [40] to entry our study, which can exclude those non-axial length high myopia. And 13 SNPs in the study of Sayaka Sasaki [37] did not include 4 of 5 SNPs in our study, except rs11664063. For rs11664063, we found the genotype and the allele frequencies of patients in our study were similar with theirs (Sayaka Sasaki [37] study: (allele frequencies) G 86.2%/A13.8%; (genotype frequencies) GG 74.3%/AG 23.9%/AA1.8%). But the genotype frequencies of controls were much more different (our study: AA 2.9%/AG 32%/GG 65%; Sayaka Sasaki [37] study: AA 3.1%/AG 22%/GG 74.9%), we think the discrepancy maybe due to the different selection criteria for control subjects in our study. Thus, population differences between the two studies may have contributed to the difference in the result.

In their study [37], they selected the13 SNPs ensuring their wide distribution over the regions ranging from exon 1 to 62, but actually the 13 SNPs didn’t reflect the relations between high myopia and all polymorphisms in *LAMA1*. Thus, they suggested more detailed SNPs analysis in *LAMA1* may be necessary for the complete screen of the entire *LAMA1* gene. While, we selected our 5 SNPs based on in-depth study of the functions of *LAMA1* SNPs and focused on those likely to alter *LAMA1* gene transcription or translation. We used the AliBaba2.1 software to predict the putative regulatory elements in the *LAMA1* promoter region. The result showed a 2,000 bp promoter region contains several putative transcription factor binding sites, such as Oct-1 (octamer-1), Sp-1 (specificity protein 1), NF-1 (nulear factor 1), C/EBPalp (CCAAT/enhancer binding protein alpha), NF-kappaB (Nuclear Factor-KappaB), AP-2alpha (transcription factor activator protein alpha), c-Jun, and AP-1 (activator protein 1). And rs2089760 just locates at a C/EBPalp element binding site (at −1,142 bp upstream from the transcription initiation) which indicates this polymorphism may influence transcriptional efficiency.

In conclusion, our results indicate that the polymorphism of rs2089760, located in the promoter region of *LAMA1*, may be associated with high myopia in Chinese population and should be investigated further.
